# Multiple sequence alignments of partially coding nucleic acid sequences

**DOI:** 10.1186/1471-2105-6-160

**Published:** 2005-06-28

**Authors:** Roman R Stocsits, Ivo L Hofacker, Claudia Fried, Peter F Stadler

**Affiliations:** 1 Interdisciplinary Centre for Bioinformatics, University of Leipzig, Haertelstraße 16-18, D-04107 Leipzig, Germany; 2 Institute for Theoretical Chemistry, University of Vienna, Währingerstraße 17, A-1090 Wien, Austria; 3 Bioinformatics Group, Department of Computer Science, University of Leipzig, Haertelstraße 16-18, D-04107 Leipzig, Germany; 4 Santa Fe Institute, 1399 Hyde Park Rd., Santa Fe NM 87501, USA

## Abstract

**Background:**

High quality sequence alignments of RNA and DNA sequences are an important prerequisite for the comparative analysis of genomic sequence data. Nucleic acid sequences, however, exhibit a much larger sequence heterogeneity compared to their encoded protein sequences due to the redundancy of the genetic code. It is desirable, therefore, to make use of the amino acid sequence when aligning coding nucleic acid sequences. In many cases, however, only a part of the sequence of interest is translated. On the other hand, overlapping reading frames may encode multiple alternative proteins, possibly with intermittent non-coding parts. Examples are, in particular, RNA virus genomes.

**Results:**

The standard scoring scheme for nucleic acid alignments can be extended to incorporate simultaneously information on translation products in one or more reading frames. Here we present a multiple alignment tool, codaln, that implements a combined nucleic acid plus amino acid scoring model for pairwise and progressive multiple alignments that allows arbitrary weighting for almost all scoring parameters. Resource requirements of codaln are comparable with those of standard tools such as ClustalW.

**Conclusion:**

We demonstrate the applicability of codaln to various biologically relevant types of sequences (bacteriophage Levivirus and Vertebrate Hox clusters) and show that the combination of nucleic acid and amino acid sequence information leads to improved alignments. These, in turn, increase the performance of analysis tools that depend strictly on good input alignments such as methods for detecting conserved RNA secondary structure elements.

## Background

Multiple sequence alignments are a crucial prerequisite for a diverse set of methods ranging from the reconstruction of phylogenies and the quantification of adaptive evolution, to the detection of conserved RNA secondary structures and protein motifs. In this contribution we present a novel alignment tool that has been developed primarily with the aim of improving multiple alignments of viral genomes. The genomes of RNA viruses often carry conserved RNA structures that perform vital functions during the life cycle of the virus. In many cases only a small part of the viral genome is functionally relevant at the level of RNA. Algorithms for the systematic search of conserved secondary structure patterns in large RNA, such as QRNA [1], alidot [2-4], RNAz [5], and RNAdecoder [6] are based on the observation that a small number of point mutations is very likely to cause large changes in the secondary structures [7]. Secondary structure elements that are consistently present in a group of sequences with less than, say, 95% average pairwise identity are therefore most likely the result of stabilizing selection, not a consequence of the high degree of sequence conservation.

A comprehensive analysis of the genomic secondary structure features using alidot is available for Picornaviridae [8], Flaviviridae [9], and Hepadnaviridae [10,11]. A preliminary survey across a large subset of the available sequence data was presented very recently [12].

The comparative approach to detect conserved RNA structures is obviously dependent upon high-quality multiple alignments: even a relative small number of alignment errors, which act like randomly placed mutations, will obscure the signals from consistent and compensatory point mutations and, hence, decrease the sensitivity of the RNA detection algorithms. While we eventually need an alignment of the genomic nucleic acid sequence, we observe that an overwhelming part of a viral genome codes for one or more proteins in one or more (overlapping) frames.

**Figure 1:**
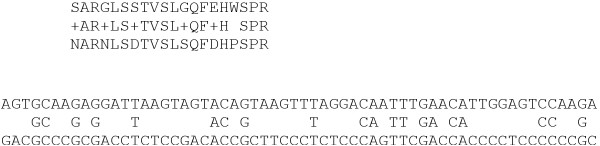
Example for the higher sequence heterogeneity on the level of nucleic acids. A hypothetical amino acid alignment on top represents a high degree of similarity. See the same sequences below on the level of nucleic acids with very low sequence similarity. The pairwise identity is only 33%, just slightly above the 25% identity expected for two random nucleic acid sequences.

In contrast to the protein sequences, which are often easily alignable, the sequence similarities are drastically reduced on the nucleic acid level due to the redundancies of the genetic code, see Fig. 1. It is desirable, therefore, to utilize the amino acid sequence when aligning *coding *nucleic acid sequences with higher sequence divergence. This is sometimes done by aligning protein sequences and subsequently back-translating to nucleic acids. In many cases, however, only a part of the sequence of interest is translated *in vivo*. In addition, there may be alternative proteins encoded in overlapping reading frames within the same nucleic acid sequences. Such overlapping reading frames are best known from viruses, including Hepatitis B [13,14], Influenza [15], and Umbraviruses [16]. Recently, however, examples have been found in prokaryotic [17,18] and even in eukaryotic genomes [19,20].

In this contribution we describe a progressive alignment tool that implements an extended scoring scheme to incorporate simultaneously information on translation products in one or more ([partly] overlapping) reading frames which allows the user to combine all information from both the nucleic acid and amino acid sequences (if any). It makes explicit use of information about overlapping open reading frames, as they occur in many functional sequences, and allows arbitrary weighting for almost all scoring parameters, in order to gain more reliable scoring at certain regions of the nucleic acid sequences where additional amino acid scoring of underlying protein sequence can be utilized.

## Implementation

The codaln program implements Gotoh's algorithm for pairwise sequence alignments with affine gap cost functions [21]. The only change compared to this standard recursive algorithm for nucleic acid sequence alignment concerns the (mis)match score *σ*(*x*_*i*_, *y*_*j*_) of position *i *from sequence *x *with position *j *from sequence *y*. Instead of taking into account only the nucleic acid letters, each position is considered as a vector containing the nucleic acid letter *and *the amino acid that would arise from translation in each of the three possible reading frames *provided *the frame in question is actually translated. Thus, we have

*σ*(*x*_*i*_, *y*_*j*_) = *β*_0_*σ*_*n*_(*x*_*i*_, *y*_*j*_) +

*β*_1_*σ*_*p*_(*π*[*x*_*i*_*x*_*i*+1_*x*_*i*+2_], *π*[*y*_*j*_*y*_*j*+1_*y*_*j*+2_]) +

*β*_2_*σ*_*p*_(*π*[*x*_*i*-1_*x*_*i*_*x*_*i*+1_], *π*[*y*_*j*-1_*y*_*j*_*y*_*j*+1_]) +     (1)

**Figure 2:**
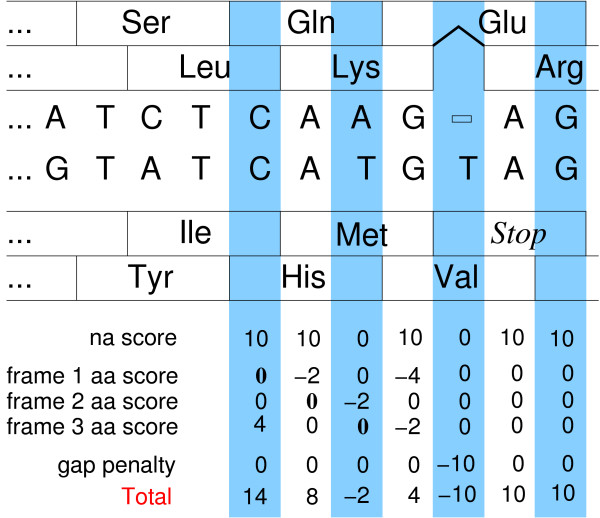
Application of the scoring model to a hypothetical alignment. Note that there are no amino acid contributions in the right hand part of the example because of the single indel that causes a frameshift. For illustration we show BLOSUM62 scores and simple scores for nucleic acids and gaps rather than the rescaled default values (His/Gln has score 0).

*β*_3_*σ*_*p*_(*π*[*x*_*i*-2_*x*_*i*-1_*x*_*i*_], *π*[*y*_*j*-2_*y*_*j*-1_*y*_*j*_])

where *π*[*uvw*] denotes the amino acid corresponding to the codon *uvw*. Here *β*_*k *_= 1, *k *= 1, 2, 3, if both *x *and *y *are translated in the *k*-th reading frame, while *β*_*k *_= 0 if the *k*-th reading frame is not actually translated in either *x *or *y *(or if one chooses to ignore a particular reading frame). *β*_0 _is the relative weight of the the nucleic acid match score, usually 1. In non-coding (untranslated) regions we therefore retain only the nucleic acid score. Fig. 2 gives an example. Further, it is possible that one gives different weights for alternative reading frames, maybe dependent upon parameters such as preferred codon usage. Default is no preference.

The score model is much simpler than the one proposed by Hein [22,23] and implemented in combat [24] and CAT [25]. In contrast to these approaches, which enforce gap lengths that are multiples of three in order to maintain the reading frame, codaln does not use special gap penalties at all. Instead, it relies on the match scores from the coding regions to guide the alignment back into the correct reading frame after a frameshift insertion or deletion. This results in an algorithm that is both faster and able to handle overlapping reading frames.

In its current implementation codaln can deal with 18 different codon tables, including the standard genetic code (default), various non-canonical tables for certain groups of organisms, and 11 distinct codon tables for mitochondrial genomes.

The codon tables link the nucleic acid triplets with their encoded amino acids. They are used both for an automatic search for start and stop codons and for translation in the scoring function; see Tab. 1.

**Table 1:** 18 codon tables can be utilized by the program for linking the nucleic acid triplets with their corresponding amino acids.

option	organism featuring this codon table
univ	universal genetic code (default)
acet	Acetabularia
ccyl	Candida cylindrica
tepa	Tetrahymena, Paramecium, Oxytrichia, Stylonychia, Glaucoma
eupl	Euplotes
mlut	Micrococcus luteus
mysp	Mycoplasma, Spiroplasma
mitocan	canonical mitochondrial code
mitovrt	Vertebrates – mitochondrial code
mitoart	Arthropods – mitochondrial code
mitoech	Echinoderms – mitochondrial code
mitomol	Molluscs – mitochondrial code
mitoasc	Ascidians – mitochondrial code
mitonem	Nematodes – mitochondrial code
mitopla	Plathelminths – mitochondrial code
mitoyea	Yeasts – mitochondrial code
mitoeua	Euascomycetes – mitochondrial code
mitopro	Protozoans – mitochondrial code

The program furthermore provides a flexible scheme for modifying the scoring model. Both amino acid and nucleic acid scores can be either taken from built-in defaults or read in from parameter files. A number of scaling factors can be specified in order to determine the relative weights of nucleic acids and/or amino acids in all the different reading frames. Tab. 2 summarizes the most important defaults. The program reads sequences in Pearson's (FASTA) format, GenBank file format, ViennaRNA format as well as completely unformatted sequences in any combination.

**Table 2:** Default scoring parameters (can be arbitrarily weighted or changed by user defined settings).

parameter	default value
protein scores	BLOSUM62 ×50
nucleic acid scores	identity 1000, else 300
gap open penalty	-1500
gap extension penalty	-300

**Remark. **The effect of the positive default score for nucleic acid mismatches is to reduce the influence of nucleic acid mismatches relative to the amino acid and gap scores.

**Figure 3:**
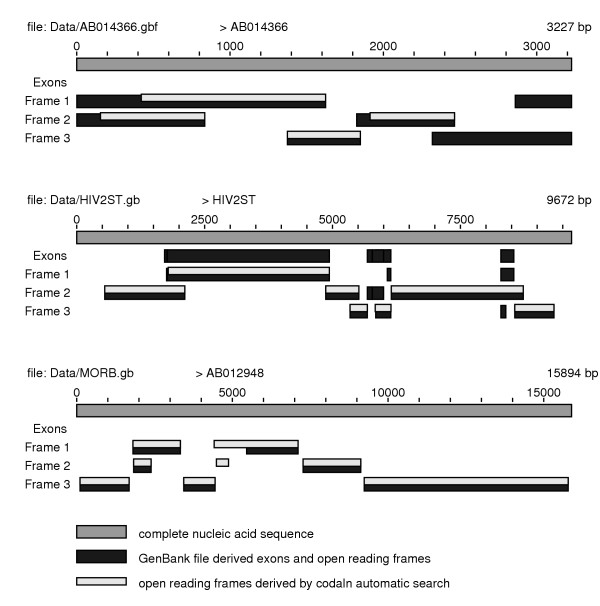
Reports on the annotated and inferred structure of the input sequences are automatically generated by codaln, respecting all user intervention.

The program uses the information about translated regions, if contained in the input file. Alternatively, codaln attempts to detect all theoretically possible open reading frames which have a user-defined minimal length. Exons and fragmented coding regions are joined, translated, and the resulting amino acid sequences are then used for the scoring function in addition to the nucleic acid sequences. The program can optionally regard a sequence as circular so that a coding region can wrap around the ends of the sequence and is still scored correctly. An intermediate output reports the structure of annotated and inferred exons and open reading frames both in a text and in PostScript format, Fig. 3. At this stage, the user can stop the process, edit the annotation file, and restart the alignment procedure with the modified annotations. The coding regions that are used for scoring can be automatically defined, user defined, modified, or eliminated. Before restarting the alignment process, codaln again provides a text and PostScript output summarizing the modified annotation. If necessary, this process can be repeated. Multiple alignments are built from the pairwise alignments using the same progressive scheme that is used e.g. by ClustalW [26]: A guide tree is inferred from the pairwise distances and determines the order in which profiles are constructed from alignments of two sequences, a sequence and a profile, or two profiles.

The profile alignments respect the full model of both nucleic acid and amino acid (mis)match scores. In the present implementation, the sequences within a profile are unweighted; it would be straightforward, however, to include a weighting scheme that takes the relative distances of the sequences into account to reduce the weight of very similar sequences, as implemented e.g. in ClustalW.

## Results

### More plausible alignments

Not surprisingly, we observe that codaln multiple alignments of coding DNA sequences have a much larger fraction of gaps with a length divisible by three than ClustalW multiple alignments. This is the desired effect of including amino acid-based scoring contributions since it reduces biologically implausible frameshifts. In itself, this is of course not a direct evidence for real improvements of multiple nucleic acid sequence alignments, but it is a good indication that, in coding regions, codaln preferentially makes insertions and deletions at the protein level.

Unfortunately, good hand-curated multiple alignments of partially coding sequences do not seem to be available, so that a systematic quantitative analysis (using, e.g., the BAliBASE tools [27]) could not be performed. Pairwise alignments of coding DNA sequences are barely distinguishable from those obtained with combat [24] provided the amino acid contributions dominate codaln's scoring function. We therefore concentrate on a qualitative assessment of codaln alignments in particular application contexts.

### Hox genes and their genomic neighborhood

*Hox *genes were first described in the fruitfly *Drosophila melanogaster*. They code for homeodomain containing transcription factors [28] and are characterized by a 60 amino-acid helix-turn-helix DNA binding homeodomain. This domain is highly conserved on the level of protein, but can be quite variable at the DNA level.

Vertebrates, in contrast to all invertebrates examined, have multiple *Hox *gene clusters that have arisen from a single ancestral cluster in the most recent common ancestor of chordates [29,30]. This ancient cluster in turn evolved through tandem gene duplications from a more ancient hypothetical protohox cluster [31].

**Figure 4:**
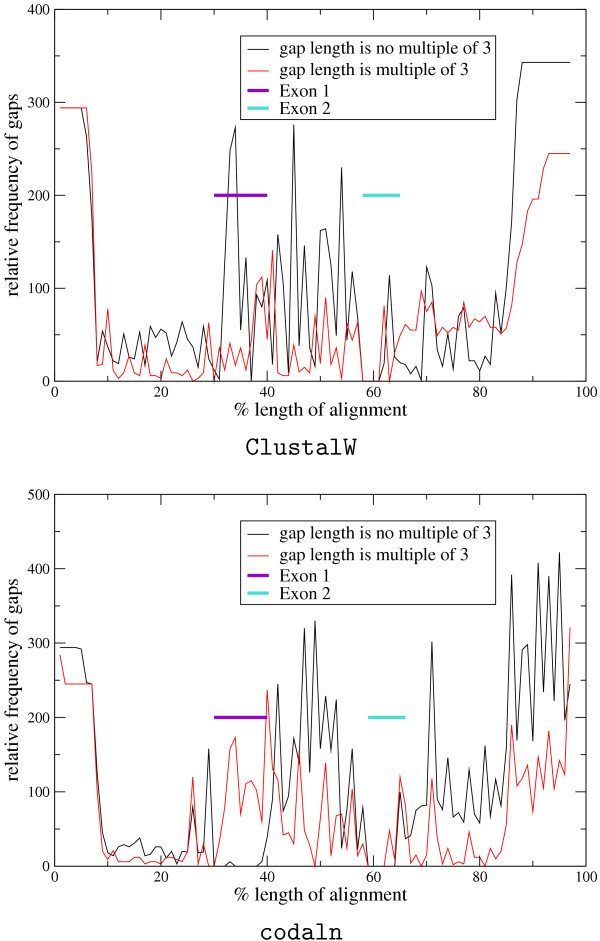
Relative distribution of gaps in an alignment of genomic *Hox4 *sequences. The alignment is essentially gap-less in *exon 2*. ClustalW (above) returns a very poor alignment of *exon 1 *in which gaps occur with a broad distribution. In contrast, codaln respects the coding region so that almost all gap lengths in this area are divisible by 3.

We applied both ClustalW and codaln to the genomic sequences at the *Hox4 *locus. *Exon 2 *of *Hox4*, which contains the homeobox, is highly conserved also on the level of nucleic acid, while *exon 1 *has a well-conserved amino acid sequence but exhibits high variability at the nucleic acid level. The non-coding sequence in the intron and the flanking sequences are highly variable. Thus, this example is a hard test case for our approach. Fig. 4 summarizes the gap lengths in the *Hox4 *alignments. A comparison of the number of gaps with a length divisible by 3 with the other gaps of other lengths is a useful indicator whether coding regions are reasonably aligned: Base triplets preferentially should not be disrupted as amino acids within a protein sequence cannot be disrupted. In this example, codaln produces 436 gaps with a length divisible by 3 (ClustalW: 330) and 797 others (ClustalW: 1113). While codaln produces a significantly higher fraction of gaps that are a multiple of 3 and correctly aligns the coding sequences in both exons, ClustalW only treats *exon 2 *correctly, which is highly conserved on the level of nucleic acids. The nucleic acid alignment for the more variable *exon 1*, in contrast, is much more divergent.

### Conserved RNA secondary structures in Levivirus genomes

Virus genomes serve as an ideal test case for a procedure that makes explicit usage of information about (overlapping) coding regions to improve the resulting alignments. Improved alignments, as we shall see, increase the sensitivity of the detection of secondary structure elements.

The members of the genus Levivirus infect eubacteria (Procarya). All members of the family Leviviridae (Levivirus and Allolevivirus) are ssRNA positive-strand viruses. The replication cycle includes no DNA stage. The virions are neither enveloped nor tailed with nucleocapsids that are isometric, 24–26 nm in diameter. The total genome length is 3466 up to 4276 nucleotides depending on type of strain. Most Levivirus species have four (partly) overlapping genes, while some exceptions exist which contain only three open reading frames [32,33].

We have investigated 8 complete genomic sequences of the Levivirus genus: The Enterobacteria phages MS2, KU1, GA, and fr. Alignments of the genomic sequences were prepared using codaln and scanned for conserved RNA secondary structures using the alidot method [3]. The results are compared to earlier studies based on ClustalW alignments [10,12].

**Figure 5:**
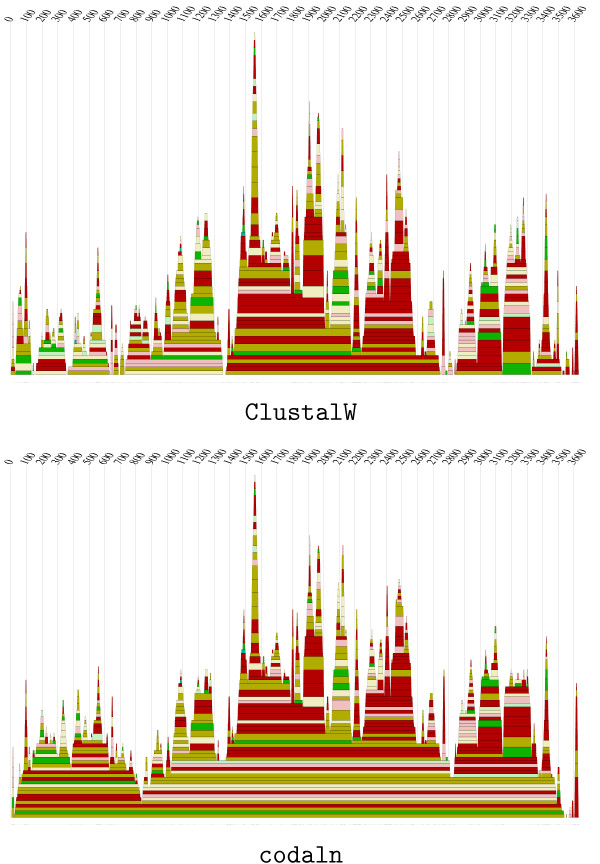
Hogeweg mountain plots of conserved RNA structures in Levivirus genomes. Above: ClustalW, below: codaln. Colors indicate the number of consistent mutations: red 1, ochre 2, green 3, turquoise 4, blue 5; Saturated colors indicate that there are only sequences that are compatible to the structure prediction. Decreasing saturation of the colors indicates 1 or 2 non-compatible sequences. The thickness of the slabs is proportional to the average frequency of the base pair in the thermodynamic equilibrium. For further details see [3].

The two different alignment processes produce results that seem to be similar at a first glance: The number of gaps and a visual interpretation of the quality of the alignment only does not already announce the significantly different results when further processing the alignments by alidot. Interestingly, the combination of codaln and alidot produces a weak signal at the basis of the Hogeweg mountain plot (see Fig. 5).

Long range interactions, so called *panhandle *structures, are known to play a role e.g. in the replication of Bunyaviridae [34] and Flaviviridae [35]. It will be interesting to see if the long-range interactions can be experimentally verified in Leviviridae as well.

**Figure 6:**
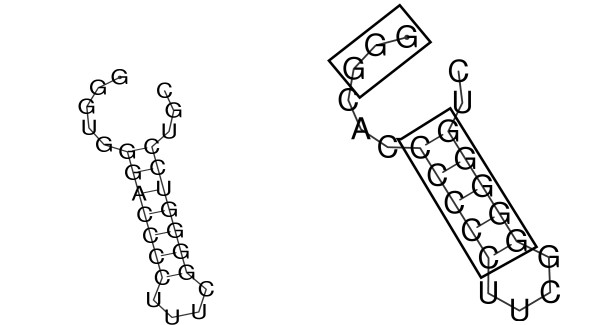
The 5'-terminal hairpin in Levivirus (left) is probably the analogon to the recognition signal site for the RNA replicase in Alloleviviruses which is well analyzed in Q*β *(right). In Q*β *the replicase amplifies RNA templates autocatalytically with high efficiency. This recognition element in Levivirus likely has a similar function.

At the 5'-terminal end of the Levivirus sequences we furthermore detect a short GC-rich hairpin(tetraloop) adjacent to an unpaired GGG element, see Fig. 6. This feature is probably the analogon to the recognition signal site for the RNA replicase in Alloleviviruses. This stem-loop-structure is well known and defined in Q*β *(Allolevivirus).

The Q*β *replicase amplifies RNA templates autocatalytically with high efficiency, and the recognition element, consisting of a hairpin and a short unpaired region at the 5'-terminus, is essential for recognition [36,37].

## Discussion

Algorithms for the the automatic detection of biologically functional secondary structure elements, such as the ones used here, are dependent upon accurate alignments. Clearly, the quality of alignments can be enhanced by including additional biological information. In the case of codaln, we make use of the information on the coding properties of a nucleic acid sequence into the alignment process. We demonstrate this in the case of alignments of the *Hox4 *genomic region which consists of non-coding regions and two coding exons, one containing the highly conserved homeodomain, while the other exon is poorly conserved on nucleic acid level. As expected, the quality of the alignment in the coding region can be increased significantly.

Virus genomes can serve as an ideal test case for a procedure that makes explicit usage of information about various (overlapping) coding regions. Above we have seen that additional conserved secondary structure elements become detectable with the improved alignment. Leviviruses are, despite their short genome, a quite complex example. The sequences are at least in part highly divergent at the nucleic acid level, so that the information on the coding sequences in codaln significantly improves the quality of the alignment. Using codaln instead of a purely nucleotide-based alignment program, we found a putative recognition signal site, analog to the one for the RNA replicase in Alloleviviruses.

## Conclusion

The codaln program was specifically developed for applications to genomic sequences of RNA viruses with their partially overlapping reading frames and untranslated regions. The *Hox *gene example shows, however, that codaln is also applicable to other partially coding sequence data. The recent discovery of ORFs that overlap with different reading directions [38-40] suggest to extend codaln to such cases as well. Our framework makes such an extension straightforward.

## Availability and requirements

C source code and documentation may be downloaded from  or .

### Hox4 data sources

The *Hox4 *sequences are taken from GenBank for *Homo sapiens *(HsA join(AC004080.2rc+AC010990 [201-6508]rc+AC004079 [75001-end]rc) [125253 126761], HsB NT_010783 [5306154 5309021]rc, HsC NT_009563 [586220 584941]rc, HsD NT_037537 [4224691 4225996]), *Mus musculus *(MmA NT_039343 [3862302 3864022]rc, MmB AC011194 [114551 116043], MmC NT_028016 [137212 139414], MmD AC015584 [164151 165456]), and *Morone saxatilis *(MsA AF089743 [29109 30386]). For *Danio rerio *the sequences are retrieved both from the web server of the *Danio rerio Sequencing Project *[41] and GenBank (DrAa AC107365rc [61628 62827], DrBa AL645782.2 [33537 35809], DrCa ctg23.10700001-10870000 [75679 77005]rc, DrD ctg13407.19000-191000 [61789 63580]rc).

rc = reverse complement; sequence intervals are listed in brackets.

## Authors' contributions

RS implemented the algorithm, RS and CF performed quantitative comparisons, ILH and PFS conceived this study. All four authors closely collaborated in writing the manuscript.
